# Sleep Disorders and Optic Neuritis Risk in Multiple Sclerosis: A Retrospective Cohort Study

**DOI:** 10.7759/cureus.77144

**Published:** 2025-01-08

**Authors:** Majd A AbuAlrob, Abdallah Hussein, Ahmad Hassan

**Affiliations:** 1 Neurology, Hamad General Hospital, Doha, QAT; 2 Internal Medicine, Virtua Our Lady of Lourdes Hospital, Camden, USA; 3 Internal Medicine, Thomas Jefferson University Hospital, Cherry Hill, USA

**Keywords:** multiple sclerosis, optic neuritis, propensity score matching, risk factors, sleep disorders

## Abstract

This retrospective cohort study investigates the relationship between sleep disorders and the risk of developing optic neuritis in patients with multiple sclerosis (MS). Utilizing data from the TriNetX Global Collaborative Network, we analyzed two matched cohorts as follows: MS patients with documented sleep disorders (n = 48,995) and those without (n = 48,995). Propensity score matching ensured balance in baseline characteristics, minimizing confounding factors.

Our findings revealed a significantly increased incidence of optic neuritis among MS patients with sleep disorders, with a 3.0% absolute risk increase compared to the control cohort. This association corresponded to an odds ratio of 1.532, indicating a substantial elevation in risk. Kaplan-Meier survival analysis further supported this link, demonstrating a 31.5% higher hazard of developing optic neuritis over time in patients with sleep disorders. These results emphasize the importance of integrating sleep health evaluations into MS management protocols. Proactively addressing sleep disorders may reduce the occurrence of optic neuritis, potentially improving the quality of life and long-term outcomes for MS patients. Future studies should explore the impact of specific sleep disorder subtypes and investigate the underlying biological mechanisms to better understand and mitigate this risk. This work underscores the broader implications of sleep health in chronic disease management and the potential for targeted interventions to alleviate disease burden.

## Introduction

Optic neuritis is a common and often early manifestation of multiple sclerosis (MS), a disabling neurological condition characterized by inflammation and demyelination of the optic nerve. This condition frequently results in significant visual impairment, including blurred vision, pain with eye movement, and reduced contrast sensitivity [1]. Such visual dysfunction can profoundly affect a patient’s quality of life, limiting daily activities, reducing independence, and impacting overall well-being. Moreover, optic neuritis has implications for the progression and long-term outcomes of MS, underscoring its clinical importance [2].

While the pathophysiology and risk factors of optic neuritis are well-studied, the potential influence of comorbid conditions, such as sleep disorders, remains unclear [3]. Sleep disturbances, including insomnia, sleep apnea, and hypersomnia, are highly prevalent in MS patients and are associated with worsened neurological symptoms, cognitive impairment, and immune dysregulation [4]. These conditions are linked to systemic inflammation and circadian rhythm disruptions, mechanisms that could intersect with the autoimmune and inflammatory processes central to MS. Consequently, there is a reasonable basis to hypothesize that sleep disorders might elevate the risk of optic neuritis in this population [5,6]. Despite this theoretical link, evidence exploring the relationship between sleep health and optic neuritis risk is limited.

Although some studies suggest that sleep disturbances may not directly cause optic neuritis, they emphasize the primary role of MS-specific autoimmune and inflammatory mechanisms in its development [7]. However, systemic inflammation and circadian rhythm disruptions related to sleep disorders could contribute indirectly to these processes, warranting further investigation [8].

This study examines the association between sleep disorders and the incidence of optic neuritis in MS patients using a retrospective cohort analysis of real-world data from the TriNetX Global Collaborative Network. By leveraging robust propensity score matching to control for confounding variables, the study provides a comprehensive analysis of this relationship. The findings aim to clarify the impact of sleep health on MS outcomes and inform potential strategies to reduce the risk of optic neuritis.

## Materials and methods

Study design and data source

This study employed a retrospective observational design, utilizing data from the TriNetX global federated health research network. TriNetX aggregates de-identified electronic medical records from 142 healthcare organizations worldwide, providing a robust platform for large-scale comparative cohort analyses. The analysis was conducted on data extracted on December 21, 2024, with cohorts defined and statistical analyses performed through the platform.

Cohort definition

The study defined two cohorts of MS patients aged ≥18 years based on the presence or absence of sleep disorders. Cohort 1 (exposed) included patients with a diagnosis of sleep disorders, such as insomnia (International Classification of Diseases {ICD}-10: G47.0) or sleep apnea (ICD-10: G47.3). Cohort 2 (control) consisted of patients without any diagnosis of sleep disorders or related conditions.

Exclusion criteria included a history of cerebral infarction, nontraumatic intracerebral hemorrhage, or intracranial injury to minimize confounding effects. Propensity score matching (PSM) was applied at a 1:1 ratio, balancing baseline demographics and clinical characteristics between cohorts. After matching, each cohort consisted of 48,995 patients.

Cohort sizes before and after matching

Cohort sizes before matching and the adjustments applied through PSM are presented in Table 1. Matching ensured balanced baseline characteristics, enhancing the validity of subsequent comparisons.

**Table 1:** Cohort sizes before and after propensity score matching. This table shows the number of patients in each group (MS with sleep disorders and MS without sleep disorders) before and after propensity score matching. Matching was performed to balance baseline demographic and clinical characteristics between the two groups. MS: multiple sclerosis

Cohort	Before matching (N)	After matching (N)
MS with sleep disorders	55,862	48,995
MS without sleep disorders	265,554	48,995

Index event and time window

The index event was defined as the first recorded diagnosis of MS. Outcomes analysis began one day after the index event and included all subsequent records. Patients with an index event occurring more than 20 years before the analysis date were excluded to ensure data relevance and reduce potential bias from outdated records. This exclusion criterion was based on minimizing variability in long-term diagnostic and treatment practices.

Outcomes

The primary outcome of the study was the incidence of optic neuritis, identified by diagnostic codes for optic neuritis (ICD-10: H46) or unspecified visual loss (ICD-10: H54.7). The analyses included the calculation of risk metrics such as risk, risk ratio, odds ratio, and risk difference. Additionally, Kaplan-Meier survival analysis was conducted to evaluate survival probabilities and hazard ratios for the development of optic neuritis. Frequency analysis was also performed to assess the number of optic neuritis instances per patient.

Statistical analysis

Comparisons between cohorts were conducted using descriptive statistics (means and standard deviations for continuous variables; percentages for categorical variables) and adjusted for confounders via PSM. Kaplan-Meier survival analysis, incorporating log-rank tests, evaluated survival differences between groups. Statistical significance was set at p < 0.05. These methods ensured a rigorous examination of the relationship between sleep disorders and optic neuritis in MS patients.

## Results

Cohort sizes and matching

**Figure 1:**
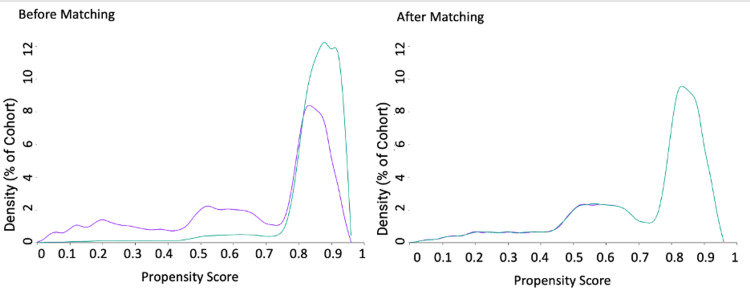
Propensity score distribution before and after matching. This figure depicts the distribution of propensity scores for MS patients with and without sleep disorders before and after matching. The overlap in distributions after matching demonstrates successful alignment of baseline characteristics.

A total of 55,862 MS patients with sleep disorders and 265,554 MS patients without sleep disorders were identified. After propensity score matching (PSM), both groups contained 48,995 patients each, ensuring comparability in baseline characteristics. The number of patients before and after matching is detailed in Table 1. The propensity score distribution, illustrating successful matching, is shown in Figure 1.

Baseline characteristics

Prior to matching, there were significant differences in demographic and clinical variables between the two groups. For example, patients with sleep disorders were generally older and had higher rates of comorbidities like hypertension and diabetes. After matching, these differences were minimized, as demonstrated in Table 2 for demographic characteristics and Table 3 for clinical characteristics. The successful alignment of baseline variables highlights the robustness of the matching process.

**Table 2:** The demographic characteristics of the two cohorts, including age, gender, race, and ethnicity. Continuous variables are presented as means ± standard deviations (SD), while categorical variables are presented as counts (n) and percentages (%). P-values were derived using independent t-tests for continuous variables and chi-square tests for categorical variables. Standardized differences are provided as a measure of effect size, with values >0.1 suggesting meaningful differences between the cohorts. Note: The total number of patients (48,995) includes individuals who did not specify their gender or identify as male or female. These patients are not included in the male (n = 34,968) or female (n = 12,314) counts but are accounted for in the overall total.

Characteristic	MS with sleep disorders (n = 48,995)	MS without sleep disorders (n = 48,995)	p-Value	Standardized differences
Age (years)				
Current age (mean ± SD)	57.7 ± 14.0	57.8 ± 14.9	0.309	0.006
Age at index (mean ± SD)	51.2 ± 13.8	51.5 ± 14.7	0.009	0.017
Gender n (%)				
Female	34,968 (71.4%)	34,801 (71.0%)	0.239	0.008
Male	12,314 (25.1%)	12,371 (25.2%)	0.675	0.003
Race, n (%)				
Black or African American	5,514 (11.3%)	5,675 (11.6%)	0.106	0.010
White	34,197 (69.8%)	34,458 (70.3%)	0.069	0.012
American Indian/Alaska Native	123 (0.3%)	83 (0.2%)	0.005	0.018
Asian	534 (1.1%)	495 (1.0%)	0.222	0.008
Native Hawaiian/Pacific Islander	388 (0.8%)	403 (0.8%)	0.592	0.003
Unknown race	6,915 (14.1%)	6,611 (13.5%)	0.005	0.018
Other race	1,324 (2.7%)	1,270 (2.6%)	0.283	0.007
Ethnicity, n (%)				
Hispanic or Latino	2,379 (4.9%)	2,460 (5.0%)	0.232	0.008
Not Hispanic or Latino	32,122 (65.6%)	32,163 (65.6%)	0.783	0.002
Unknown ethnicity	14,494 (29.6%)	14,372 (29.3%)	0.393	0.005

**Table 3:** Diagnosis characteristics of study cohorts. The prevalence of selected clinical diagnoses within the two cohorts is presented in the table. Data are shown as counts (n) and percentages (%). P-values were calculated using chi-square tests for differences in proportions between the cohorts. Standardized differences are included to indicate the magnitude of differences, with values >0.1 considered significant. Conditions are classified according to their ICD-10 codes for clarity and consistency. ICD: International Classification of Diseases

Condition	MS with sleep disorders (n = 48,995)	MS without sleep disorders (n = 48,995)	p-Value	Standardized differences
Diabetes mellitus (E08-E13), n (%)	4,936 (10.1%)	4,656 (9.5%)	0.003	0.019
Essential hypertension (I10), n (%)	12,780 (26.1%)	13,385 (27.3%)	<0.001	0.028
Nicotine dependence (F17.200), n (%)	3,981 (8.1%)	3,996 (8.2%)	0.861	0.001
Overweight/obesity (E66), n (%)	7,085 (14.5%)	6,282 (12.8%)	<0.001	0.048
Heart disease, unspecified (I51.9), n (%)	398 (0.8%)	294 (0.6%)	<0.001	0.025

Risk of optic neuritis

**Figure 2:**
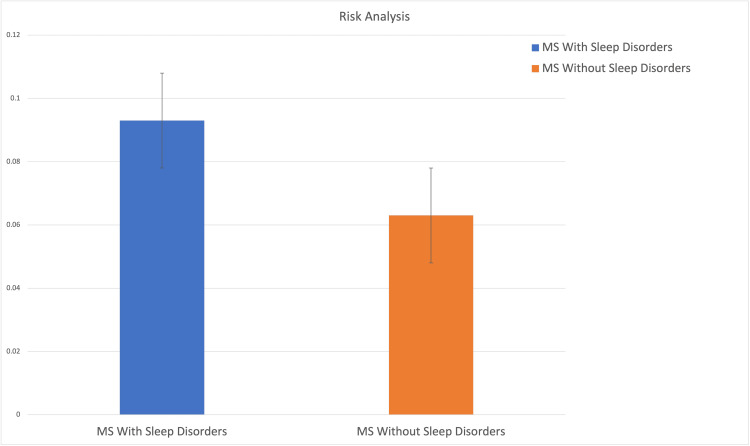
Risk analysis of outcomes in exposed (blue) and control (orange) cohorts. The bar heights represent the proportion of patients with the specified outcome within each cohort. The exposed cohort demonstrates a higher risk compared to the control cohort, reflecting the calculated risk difference. This visual provides a clear comparative analysis of outcome probabilities across the two groups.

MS patients with sleep disorders had a significantly higher incidence of optic neuritis compared to those without sleep disorders. After matching, 9.3% of the sleep disorder group developed optic neuritis versus 6.3% of the group without sleep disorders. This equates to an absolute risk difference of 3% (95% CI: 2.7-3.4), with a risk ratio of 1.483 and an odds ratio of 1.532. These results are summarized in Table 4. A visual comparison of the incidence rates is provided in Figure 2, highlighting the increased likelihood of optic neuritis in the sleep disorder group.

**Table 4:** Comparison of study outcome risks across cohorts. Patients with and without sleep disorders are presented with their respective total number of patients, outcomes, and risk. Measures of risk difference, risk ratio, and odds ratio are also reported. "Not applicable" indicates that z-statistics and p-values were not calculated for risk ratio and odds ratio.

Cohort	Patients in cohort (n)	Patients with outcome (n)	Risk	Measure	p-Value	95% CI	z-statistic	p-Value
MS with sleep disorders	48,995	4,546	0.093	Risk difference	0.03	0.027, 0.034	17.663	<0.001
MS without sleep disorders	48,995	3,066	0.063	Risk ratio	1.483	1.419, 1.550	Not applicable	Not applicable
-	-	-	-	Odds ratio	1.532	1.461, 1.607	Not applicable	Not applicable

Kaplan-Meier survival analysis

The Kaplan-Meier survival analysis revealed a lower probability of remaining optic neuritis-free in MS patients with sleep disorders. At the end of the observation period, the survival probability was 79.44% in the sleep disorder group compared to 85.86% in the non-sleep disorder group. The hazard ratio of 1.315 (95% CI: 1.256-1.377, p < 0.001) indicates an accelerated progression to optic neuritis in patients with sleep disorders. The detailed survival probabilities and hazard ratios are shown in Table 5.

**Table 5:** Kaplan-Meier survival analysis outcomes. The table includes the number of patients in each cohort, the survival probability at the end of the time window, and the hazard ratio with 95% confidence intervals. The log-rank test evaluates the statistical significance of differences in survival between cohorts.

Metric	Exposed cohort (n = 48,995)	Control cohort (n = 48,995)	Statistic/notes
Patients with outcome (n)	4,546	3,066	-
Median survival (days)	-		Not reported
Survival probability at end of time window	79.44%	85.86%	-
Log-rank test (χ²)	-		138.212
Log-rank test p-Value	-		<0.001
Hazard ratio	1.315	-	Compared to control cohort
95% Confidence interval	(1.256, 1.377)	-	

**Figure 3:**
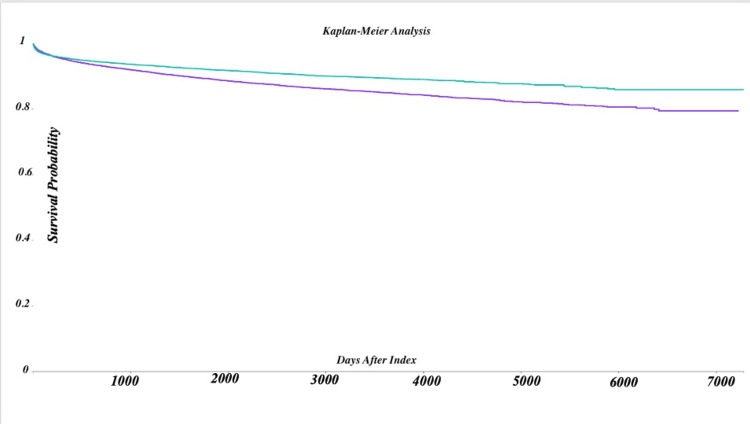
Kaplan-Meier survival curve for exposed and control cohorts. Curve colors show the following: teal line - survival probability of the exposed cohort (patients with sleep disorders); purple line - survival probability of the control cohort (patients without sleep disorders). The X-axis shows days after the index event, the time elapsed since the index event for each cohort. The Y-axis shows survival probability, the proportion of patients surviving over the observed time window. Key observations: the teal curve (exposed cohort) indicates a lower survival probability over time compared to the purple curve (control cohort), highlighting the differences between the two groups.

Frequency of optic neuritis episodes

Patients with sleep disorders not only had a higher risk of developing optic neuritis but also experienced it more frequently. The mean number of episodes in the sleep disorder group was 6.443 ± 18.139, compared to 4.240 ± 9.980 in the non-sleep disorder group (p < 0.001). The median frequency was two episodes for both groups, but the higher mean and variance in the sleep disorder group suggest increased severity or recurrence. These findings are detailed in Table 6.

**Table 6:** Analysis of the number of instances for the exposed and control cohorts. The mean number of instances is reported alongside statistical test results, including the t-statistic, degrees of freedom, and p-value, to assess the significance of differences between cohorts.

Metric	MS with sleep disorders (n=48,995)	MS without sleep disorders (n=48,995)	Statistics
Patients in cohort (n)	48,995	48,995	-
Patients with outcome (n)	4,546	3,066	-
Mean number of instances	6.443	4.24	-
Test statistic (t)	-	-	6.129
Degrees of freedom (df)	-	-	7,610
p-Value	-	-	<0.001

## Discussion

This study provides robust evidence that sleep disorders significantly increase the risk of optic neuritis among patients with multiple sclerosis (MS). Leveraging a large and diverse cohort through propensity score matching (PSM), we observed that MS patients with sleep disorders were approximately 1.5 times more likely to develop optic neuritis compared to those without sleep disorders (risk ratio: 1.483, odds ratio: 1.532). Furthermore, Kaplan-Meier survival analysis demonstrated that optic neuritis-free survival probability was substantially lower in the sleep disorder group, with a hazard ratio of 1.315. These findings suggest that sleep disturbances may accelerate MS-related complications, particularly optic neuritis, and underscore the need for integrated management of sleep health in MS care [1].

Chronic sleep disorders, including insomnia and sleep apnea, exacerbate systemic inflammation and immune dysregulation, mechanisms pivotal to MS pathophysiology. By amplifying neuroinflammatory cascades that underlie demyelination, sleep disturbances may increase susceptibility to optic neuritis [9]. The recurrence and higher frequency of optic neuritis episodes observed in the sleep disorder group reinforce the notion that sleep quality not only influences disease onset but also contributes to the progression and severity of MS-related complications [3]. These findings align with previous studies demonstrating a bidirectional relationship between sleep disturbances and neuroinflammation, while also offering new insights into the specific impact on optic neuritis.

Our study stands out by focusing on optic neuritis as a distinct clinical outcome. Unlike broader research on sleep disorders and MS progression or quality of life, this targeted approach provides novel evidence on how sleep disturbances may exacerbate a critical marker of MS disease activity. Optic neuritis, which often precedes severe neurological disability, is particularly important in assessing disease progression, and our analysis of a large, diverse cohort strengthens the generalizability of these findings [10]. This evidence highlights the need for routine evaluation of sleep disorders in MS patients, a practice that could enable earlier intervention and reduce the risk of optic neuritis and other complications [11].

The clinical implications of these findings are substantial. Despite the high prevalence of sleep disturbances in MS, these conditions remain underdiagnosed and undertreated. Incorporating validated screening tools for conditions such as insomnia and sleep apnea into routine MS care could mitigate the risk of optic neuritis and improve overall disease management. Interventions like cognitive-behavioral therapy for insomnia and continuous positive airway pressure (CPAP) therapy for sleep apnea represent promising strategies for reducing neuroinflammatory burden and enhancing patient outcomes. These approaches may also alleviate overlapping symptoms, such as fatigue and cognitive dysfunction, further contributing to better quality of life and disease management [12]. Personalized treatment plans that include sleep health could allow for better risk stratification, ensuring more targeted therapeutic interventions for high-risk individuals [13]. Additionally, addressing sleep disturbances may enhance adherence to MS therapies and improve patient satisfaction with care, amplifying the overall effectiveness of disease management strategies [14]. Recognizing the complex interplay between sleep and MS, multidisciplinary approaches to care that involve neurologists, sleep specialists, and mental health professionals may offer the most comprehensive benefits [15].

While this study benefits from the use of a large, real-world dataset derived from the TriNetX network, its retrospective design introduces limitations. Reliance on pre-existing medical records may lead to biases related to data accuracy and completeness. Potential misclassification of sleep disorders or optic neuritis diagnoses could influence the results. Furthermore, although PSM effectively balanced measured baseline characteristics, unmeasured confounders such as physical activity, diet, and adherence to MS therapies were not accounted for. These factors could interact with both sleep quality and disease progression, underscoring the need for future research employing prospective designs with more comprehensive data collection. Additionally, the study focused exclusively on optic neuritis as an outcome, leaving the relationship between sleep disturbances and other MS-related complications unexplored. Future studies should address this gap to provide a broader understanding of sleep health in MS [9].

Future research should prioritize investigating the biological mechanisms linking sleep disturbances to neuroinflammation and optic neuritis in MS. Biomarkers such as inflammatory cytokines, oxidative stress indicators, and measures of myelin integrity could elucidate these pathways. Randomized controlled trials are also needed to evaluate the efficacy of sleep disorder treatments, such as CPAP and cognitive-behavioral therapy, in reducing optic neuritis risk and improving MS outcomes. Exploring interactions between sleep disturbances and other MS comorbidities, including anxiety and depression, may further clarify how these factors compound neuroinflammatory processes and disease progression. Longitudinal studies tracking the temporal relationship between sleep quality and optic neuritis could identify high-risk subgroups, enabling more targeted and effective interventions [10-12].

By identifying a significant association between sleep disorders and the risk of optic neuritis, this study highlights the importance of addressing sleep health as a core component of MS management. Incorporating sleep assessments into routine care, alongside targeted interventions, could reduce the burden of optic neuritis and improve outcomes for MS patients. Building on these findings through future research will further elucidate the mechanisms at play and refine strategies to integrate sleep health into comprehensive MS management.

## Conclusions

This study highlights the significant association between sleep disorders and increased risk of optic neuritis in MS patients, emphasizing the critical need for integrated management of sleep health in MS care. By addressing sleep disturbances, clinicians may not only mitigate the risk of optic neuritis but also improve the overall quality of life and long-term prognosis for MS patients. Future research should continue to explore the complex interplay between sleep, neuroinflammation, and disease progression, paving the way for personalized and comprehensive treatment approaches.
